# Applying machine learning to high-dimensional proteomics datasets for the identification of Alzheimer’s disease biomarkers

**DOI:** 10.1186/s12987-025-00634-z

**Published:** 2025-03-03

**Authors:** Christoffer Ivarsson Orrelid, Oscar Rosberg, Sophia Weiner, Fredrik D. Johansson, Johan Gobom, Henrik Zetterberg, Newton Mwai, Lena Stempfle

**Affiliations:** 1 Computer Science and Engineering, Chalmers University of Technology and University of Gothenburg, Rännvägen 6b, 41296 Gothenburg, Västra Götalandsregionen Sweden; 2 Department of Psychiatry and Neurochemistry, The Sahlgrenska Academy at the University of Gothenburg, Wallinsgatan 6, 43141 Möndal, Västra Götalandsregionen Sweden; 3 Clinical Neurochemistry Lab, Clinical Neurochemistry Lab, Institute of Neuroscience and Physiology, Sahlgrenska University Hospital, Wallinsgatan 6, 43141 Möndal, Västra Götalandsregionen Sweden; 4 Department of Neurodegenerative Disease, UCL Institute of Neurology, Queen Square, London, UK; 5 UK Dementia Research Institute at UCL, UCL Institute of Neurology, Queen Square, London, UK; 6 Hong Kong Center for Neurodegenerative Diseases, Clear Water Bay, Hong Kong, China; 7 Wisconsin Alzheimer’s Disease Research Center, University of Wisconsin School of Medicine and Public Health, University of Wisconsin-Madison, Madison, WI USA

**Keywords:** Alzheimer’s disease, Proteomics, Mass spectrometry, High-dimensional data, Biomarkers, Machine learning, Feature selection

## Abstract

**Purpose:**

This study explores the application of machine learning to high-dimensional proteomics datasets for identifying Alzheimer’s disease (AD) biomarkers. AD, a neurodegenerative disorder affecting millions worldwide, necessitates early and accurate diagnosis for effective management.

**Methods:**

We leverage Tandem Mass Tag (TMT) proteomics data from the cerebrospinal fluid (CSF) samples from the frontal cortex of patients with idiopathic normal pressure hydrocephalus (iNPH), a condition often comorbid with AD, with rare access to both lumbar and ventricular samples. Our methodology includes extensive data preprocessing to address batch effects and missing values, followed by the use of the Synthetic Minority Over-sampling Technique (SMOTE) for data augmentation to overcome the small sample size. We apply linear, and non-linear machine learning models, and ensemble methods, to compare iNPH patients with and without biomarker evidence of AD pathology ($A\beta ^-T^-$ or $A\beta ^+T^+$) in a classification task.

**Results:**

We present a machine learning workflow for working with high-dimensional TMT proteomics data that addresses their inherent data characteristics. Our results demonstrate that batch effect correction has no or minor impact on the models’ performance and robust feature selection is critical for model stability and performance, especially in the high-dimensional proteomics data setting for AD diagnostics. The results further indicated that removing features with missing values produced stronger models than imputing them, and the batch effect had minimal impact on the models Our best-performing disease-progression detection model, a random forest, achieves an AUC of 0.84 (± 0.03).

**Conclusion:**

We identify several novel protein biomarkers candidates, such as FABP3 and GOT1, with potential diagnostic value for AD pathology detection, suggesting the necessity of different biomarkers for AD diagnoses for patients with iNPH, and considering different biomarkers for ventricular and lumbar CSF samples. This work underscores the importance of a meticulous machine learning process in enhancing biomarker discovery. Our study also provides insights in translating biomarkers from other central nervous system diseases like iNPH, and both ventricular and lumbar CSF samples for biomarker discovery, providing a foundation for future research and clinical applications.

## Introduction

Alzheimer’s disease (AD) is an ageing-associated neurodegenerative disorder estimated to affect around 50 million people worldwide. This number is expected to rise to 150 million in the year 2050 as life expectancy increases [1], which implies a high burden on healthcare systems. Consequently, studies aiming to understand AD better are necessary to improve early diagnosis of AD (critical for management [2]), and to understand AD drug response as well as AD pathogenesis progression [3]. A clinical AD diagnosis is made through medical history, cognitive tests, and neurological examinations [4]. Conventionally, this is combined with CSF or imaging biomarkers for AD pathology. However, a definitive diagnosis of AD can only be made with certainty through brain tissue biopsies known as pathological diagnoses [5]. Prevalent procedures focus on cerebrospinal fluid (CSF) samples collected using procedures like lumbar puncture.

Recent advances in machine learning have led to studies aimed at predicting neurodegenerative disease progression with datasets containing measurements of neuropathology and cognition [6–8]. It is challenging in these machine learning prediction and modeling studies to identify which features are most predictive of disease status. Moreover, the understanding of the interaction and relationships between these often high-dimensional features derived from pathological diagnoses is somewhat limited and remains a key area of interest in the study of AD and related neurodegenerative diseases [9–14].

Pathological diagnoses involve the examination of biomarkers, which are measurable biological sets of molecules or pathogenic processes that can indicate the presence of a particular physiological or pathological disease [15]. To improve the detection rate and treatment of AD, finding reliable biomarkers to help with early diagnosis and drug response is at the forefront of dementia-related research [3]. In 1984, it was discovered that the amyloid-$\beta$ ($A\beta$) peptide is associated with AD [16]. It is one of the primarily studied biomarkers, together with tau proteins, in AD identification [17] and is now used in clinical practice to help diagnose the condition. However, AD is characterised not only by Abeta plaque and tau tangle pathology but also by tissue reaction in the form of astrocytic and microglial activation, synaptic degeneration, blood-brain barrier injury and inflammation [18]. Finding more biomarkers for AD and related neurodegenerative diseases is important to help with diagnosis and treatment selection. CSF has long been considered the sample type of choice, since it bathes the brain and since this fluid is on the brain side of the blood-brain barrier, but recent breakthroughs in ultrasensitive measurement technologies now allow for the detection of cerebral Abeta and tau pathology through biomarker measurements in regular blood samples [19]. Current proteomics AD research is focused on finding biomarkers in alternative biological samples, such as in urine, blood, or cerebrospinal fluid obtained during diagnosis of other conditions [20].

Idiopathic Normal Pressure Hydrocephalus (iNPH) is a CSF dynamics disturbance disorder that may injure neurons, and shares symptoms with AD, such as cognitive dysfunction [21]. Furthermore, patients with iNPH may have a higher risk of developing AD, and the prevalence of AD is elevated in iNPH compared to the general population [22], however, this may be due to diagnostic access bias. Biomarkers used for classification of the AD progression continuum, $A\beta$ and tau proteins, are also prevalent in iNPH CSF samples [22]. The standard procedure of iNPH diagnosis involves the analysis of lumbar CSF samples and in some cases ventricular CSF samples for research purposes. Access to both sets of samples provides interesting and otherwise rare opportunities for studying AD. Ventricular CSF samples are collected during neurosurgery by installing a CSF diversion shunt [23] to drain excess CSF from the cerebral ventricle to an extracerebral space so that the pressure on the brain is decreased [24]. Lumbar CSF is sampled a week before neurosurgery in a relatively non-invasive procedure that can be performed with or without spinal anaesthesia [20]. The CSF samples can be analyzed via LC-MS for research processes for exploratory proteomics [24, 25]. This process yields high-dimensional datasets that can be used for further investigation.

Despite advances in proteomics and machine learning techniques [17], there remain significant challenges in the detection of AD pathology in patients with other neurological disorders. Many well-documented biomarkers for general AD progression lack validation for their predictive power in differentiating AD pathology in iNPH patients, highlighting the need for identifying novel biomarkers specific to this unique cohort. Furthermore, the scarcity of studies leveraging ventricular CSF, which has distinct proteomic characteristics compared to lumbar CSF, further underscores an underexplored research area. Lastly, challenges common in proteomics datasets, such as small cohort sizes, imbalanced, high-dimensional data with missing values and batch effect, are approached with state-of-the-art machine learning techniques.

## Materials and methods

### Materials

#### Mass spectrometry

For this study, we use previously generated mass spectrometry (MS) data obtained through Tandem Mass Tag (TMT). Tandem Mass Tag (TMT) is an isobaric labeling strategy that enables parallel multiplexing, allowing multiple samples to be processed simultaneously through a mass spectrometer. It is one of the most frequently used techniques for quantifying relative protein and peptide abundance [26]. Each prepared sample is tagged with a different isobaric chemical tag variant, and equal quantities from each sample are then pooled and run through the mass spectrometer. The first MS spectrum provides a survey scan of all ions entering the mass spectrometer, while a second MS spectrum determines the relative abundance from each sample in the pool based on their unique chemical tags [27].

#### Data characteristics

We use four datasets from a study conducted by Weiner et al. (2023). The study aimed to identify prognostic CSF biomarkers for predicting shunt responsiveness in iNPH patients. The datasets were generated using bottom-up proteomics, which involved digesting the proteins in the CSF into peptides using Trypsin, a commonly used enzyme for this purpose. The peptides were then analyzed with an MS/MS instrument, and the resulting MS/MS spectra were matched to peptide sequences using the $\hbox {Sequest}^{TM}$ search engine with UniProtKB Swiss-Prot (TaxID = 9606, Homo sapiens) as the database. Peptides were subsequently matched to proteins using Proteome Discoverer 2.5.0.400. The datasets are publicly available upon request.

**Fig. 1:**
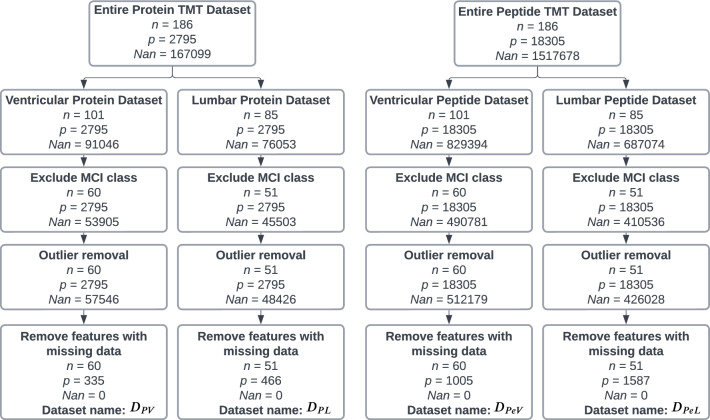
Exclusion flowchart showing the number of samples *n*, features *p* and missing values *NaN* during the data preprocessing stage

The cohort consists of 186 samples collected from 106 iNPH patients, with 85 samples from lumbar CSF fluid and 101 from ventricular CSF fluid. Both protein and peptide datasets were generated from these samples. For clarity, we refer to the datasets as $D_{PL}$, $D_{PV}$, $D_{PeL}$, and $D_{PeV}$, where *P* and *Pe* denote protein and peptide data, respectively, and *L* and *V* indicate lumbar and ventricular samples. The exclusion flowchart in Fig. 1 shows the number of subjects included in each dataset. All peptide abundances were first normalized to the reference channel (135N), which consists of the same sample and is positioned in the last channel of each TMT batch. Since the peptide abundances should be the same in each reference channel, normalizing the other samples to the reference channel’s results helps mitigate some of the batch effects. Furthermore, the dataset was median normalized to correct for variations in sample quantities. This was done by dividing each protein abundance by the median protein abundance for each sample, as defined by [26]:

1 $$\begin{aligned} \tilde{X}_{ij} = \frac{X_{ij}}{\text {median}(X_i)} \end{aligned}$$

where $X_{ij}$ is the protein abundance of protein *j* in sample *i* and $\tilde{X}_{ij}$ is the normalized protein abundance ratio. When the protein or peptide abundance is below the detection threshold of mass spectrometry (MS), it results in values that are missing not at random (NMAR) [28] in the data [29]. TMT data generally has less than 1% missing values within each batch. However, when batches are combined, additional missingness is observed because different batches may not capture the same sets of proteins and peptides. This results in a combined dataset with more apparent missingness. Thus, while each batch may have low missingness on its own, merging them reveals differences in protein capture across batches. Addressing this is crucial to ensure the conclusions drawn from machine learning models are valid and reliable. It is worth mentioning that there is randomness in peptide sampling of the mass spectrometer, another contributor to missingness.

**Table 1:** Overview of descriptive statistics of demographic features

Description	$A\beta ^-T^-$			$A\beta ^+T^-$			$A\beta ^+T^+$		
	Tot.	L	V	Tot.	L	V	Tot.	L	V
Mean Age at Biopsy	72.57	73.05	72.15	73.95	74.03	73.88	78.87	79.60	78.31
Min Age at Biopsy	53	53	53	59	64	59	64	64	64
Max Age at Biopsy	90	90	90	87	87	87	88	88	88
Std Age at Biopsy	8.10	7.96	8.28	5.70	5.27	6.10	6.23	6.52	6.20
VCI	4	2	2	0	0	0	0	0	0
AD	4	2	2	15	7	8	21	9	12
AD+VCI	4	2	2	4	2	2	2	1	1
Suspected AD	0	0	0	6	2	4	0	0	0
Genetic outlier	0	0	0	1	0	1	0	0	0
Male	54	25	29	45	19	26	14	7	7
Female	34	16	18	30	15	15	9	3	6

Descriptive statistics of demographic features categorized by tissue groups and divided into lumbar (L) and ventricular (V) CSF data. Rows 5-9 describe other clinical comorbid conditions of the patients where VCI is vascular cognitive impairment

Table 1 describes the descriptive statistics of the dataset’s demographic features. The patients of the $A\beta ^+T^+$ tissue group have a greater mean age than the other groups. All patients in this group have a clinical AD diagnosis, and one has a vascular cognitive impairment (VCI). Further, there are a few patients in the $A\beta ^-T^-$ group that have a clinical AD or AD and VCI diagnosis, but no pathological lesions were found in the brain samples. This suggests that these patients may have a different neurodegenerative disorder that is not AD (Table 2).

**Table 2:** Missing Values in TMT Batches

Dataset	Within batch missingness (N)	Combined batch missingness (%)
$D_{PL}$	0	$32.01\%$
$D_{PV}$	0	$32.25\%$
$D_{PeL}$	67	$44.20\%$
$D_{PeV}$	45	$44.89\%$

When the batches are combined, the missingness increases to $32.01\%$ and $32.25\%$ in $D_{PL}$ and $D_{PV}$, respectively. The peptide dataset $D_{PeL}$ has 67 missing values total within each batch, and $D_{PeV}$ has 45 missing values. When batches are combined, there is a missingness of $44.20\%$ for $D_{PeL}$ and $44.89\%$ for $D_{PeV}$. There is no noticeable difference between missing variables between lumbar and ventricular CSF samples.

We now turn our focus to the progression outcomes in the cohort, highlighting the prognostic indicators from biopsy statuses.

#### Progression outcomes

The cohort samples were divided into three biopsy status groups: $A\beta ^-T^-$, $A\beta ^+T^-$, and $A\beta ^+T^+$. These groups describe the presence of pathological lesions, i.e. $A\beta$ plaques and tau-tangles, that have been found in each brain sample taken from the frontal cortex and were acquired during CSF shunting. The $A\beta ^-T^-$ group indicates no pathology, $A\beta ^+T^-$ represents an earlier disease stage, and $A\beta ^+T^+$ represents a later disease stage. Domain knowledge recommends excluding the $A\beta ^+T^-$ tissue group to achieve higher generalization of the results when predicting the binary task of subjects developing from $A\beta ^-T^-$ to $A\beta ^+T^+$ status.

### Methods

#### Data preprocessing

**Fig. 2:**
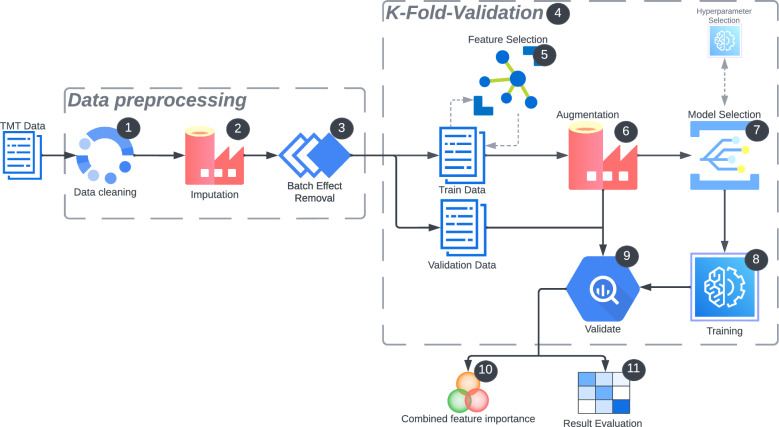
Overview of the machine learning workflow used in the project, highlighting key steps. Step 1 removes invalid data, outliers, and features with exercise missing values. Step 2 imputes data through MICE or minimum imputation. Step 3 utilizes ComBat, removing the batch effect. Step 4 partitions the data into five folds before the feature selection phase of step 5 . This is done to reduce the common risk of data leakage [36], as performing the k-fold partitioning after the feature selection would result in testing on previously seen data points. Step 6 augments synthetic data through SMOTE. Steps 7, 8, and 9 include optimizing hyperparameters, training, and evaluating the models. Steps 10 and 11 involve evaluating the results and potential biomarkers suggested by the models

Our machine learning pipeline begins with data preprocessing to transform the data into a format suitable for machine learning algorithms. This preprocessing involves removing outliers due to measurement errors and addressing missing values (Steps 1-6 in Fig. 2). The process begins with data cleaning (1), where raw data is prepared by removing inconsistencies and outliers, such as erroneous measurements equal to zero or infinity. This is followed by imputation (Step 2), where missing values are handled to ensure complete datasets, as most machine learning algorithms cannot process incomplete data effectively. Two imputation techniques, Multiple Imputation by Chained Equations (MICE) and minimum imputation, were used. For MICE, Scikit-learn’s IterativeImputer with the BayesianRidge estimator was employed, imputing the data five times independently with randomly drawn seeds for 30 iterations each. The five imputed datasets were then pooled into one by averaging them column-wise. To implement minimum imputation, we used SampMin [30] due to its computational efficiency and effectiveness. SampMin imputes missing values with the lowest observed value for each feature, addressing measurement errors during the MS phase where peptides fall below the minimum observable threshold. Next, data transformation and normalization are conducted, including batch effect removal (3), to ensure consistency and comparability across the dataset. For batch effect correction, we used the ComBat method [31], which adjusts the data by estimating location and scale parameters using an Empirical Bayes method. We implemented this using the Python library pyComBat by [32] to effectively remove batch effects. Having prepared the data with these three steps, we proceeded to define our prediction models and learning objectives.

#### Prediction models and learning objectives

First, we predicted the change in pathological diagnosis (change/no change) from $A\beta ^-T^-$ to $A\beta ^+T^+$ relative to baseline on the protein level (task A), differentiating between lumbar (task A1) and ventricular (task A2) levels. Secondly, we used peptide data to predict the change in diagnosis (task B), treating it also as a binary classification problem for lumbar (task B1) and ventricular data (task B2). For each task, we considered both linear and non-linear estimators as well as ensemble methods. Specifically, we used XGBoost (XGB) [33], Logistic Regression (LR) [34], and Random Forest (RF) [35]. These widely used and well-studied machine learning models are advantageous because they typically require less data than neural networks to perform effectively. LR and RF were implemented using the sci-kit learn library and XGBoost through the XGBoost library. In addition to running all methods individually, we used ensemble methods for their ability to improve prediction accuracy and robustness. The hyperparameter ranges for each model are shown in the Appendix Table 7. With the modeling tasks clearly defined, we next describe how we selected and validated these predictive models.

#### Model selection

In this work, we were primarily interested in evaluating how well machine learning models perform for previously unseen subjects. To obtain an unbiased estimate of out-of-sample performance, we utilized sample splitting and *k*-fold cross-validation (Steps 4 - 11 in Fig. 2). This approach involves partitioning the dataset into *k* equal-sized folds, each serving as a distinct validation set, with the model trained and evaluated *k* times. We set *k* = 5, as it provided the highest stability across each validation set. Each fold takes turns as the validation set, while the remaining data serves as the training set, effectively reducing overfitting [36]. The average performance was estimated by averaging the results across the *k* folds [37] to balance bias and variance. Having established our cross-validation framework for model selection, we also needed a feature selection strategy to identify the informative feature sets from the high-dimensional full data feature sets. In high-dimensional statistics, the relationship between the number of variables (*p*) and the number of observations (*n*) is crucial. Traditional statistical methods are designed under the assumption that $n > p$. When $p > n$, these methods often fail or underperform [38]. Reducing the feature space (*p*) is essential to extract meaningful insights from high-dimensional data.

Ensemble techniques can also be used for feature selection [39] where multiple feature selectors on the training data identify the best *k* features. These feature subsets are then aggregated using various methods such as thresholding, ranking, intersection, or union. In this study, the union aggregation method [39] was employed, combining the selected features from four distinct models. The four models used for feature selection were Lasso [34], LR, RF, and XGB. The Sklearn RFE() function was used to iteratively remove the *m* least important features from *p* until *k* features remained. Various values of *k* were examined during the modeling stage.

The stability of this ensemble feature selection method is important. For reproducibility and reliability, especially with biomarkers, it is crucial to select the same features deterministically. The stability of a feature selection algorithm reflects its robustness in producing consistent feature preferences from training data drawn from the same distribution [40]. Since feature selection is performed on separate training data in each k-fold, a higher number of matching features across each k-fold indicates greater stability.

#### Data augmentation

To enhance the data quality and diversity, thereby improving the robustness and generalizability of the machine learning models, data augmentation (Step 6) was also performed. The Synthetic Minority Over-sampling Technique (SMOTE) [41] was used. SMOTE works by generating synthetic examples for the minority class by interpolating between existing examples. This helps to balance the class distribution and prevent the models from being biased towards the majority class. To retrieve optimal hyperparameters, both GridSearchCV(REF) and BayesSearchCV(REF) [42] were used. A hyperparameter search was performed within each of the *k* folds, resulting in optimized models for each *k*-fold, without risking overfitting. Hyperparameters were tuned using BayesSearchCV from the scikit-optimize library, utilizing Bayesian Optimization. This method uses a surrogate model to represent the search space and finds parameters that maximize the scoring function. Other algorithms considered for hyperparameter tuning included GridSearchCV and RandomizedSearchCV. Recent studies have shown that random search is more efficient than grid search because it focuses on more impactful dimensions [43].

#### Model evaluation

To ensure robust and consistent evaluation, we used different five-fold cross-validation splits across 10 iterations (see steps 10 - 11 in the pipeline). The final performance was given by the average test score across these repetitions, resulting in 50 held-out test score measures from models with potentially different hyperparameters. This average score and its standard deviation indicate the expected quality of a model trained on a new, similarly-sized sample and evaluated on a held-out, similarly-sized sample.

The classification models were evaluated using a combination of metrics for a comprehensive assessment, especially for imbalanced and small-sized proteomics datasets [44, 45]. We used the weighted $F_1$ score, which averages precision and recall, accounting for both false positives and false negatives and is preferred for imbalanced class distributions [46]. Accuracy, defined as the ratio of correct predictions to total predictions, can be misleading in imbalanced datasets and is less appropriate for small sample sizes. Balanced accuracy, the arithmetic mean of sensitivity and specificity, is more suitable in these cases [44]. Area Under the Curve (AUC) assesses a classifier’s ability to distinguish between classes across various thresholds, with higher AUC values indicating better performance [47]. The Matthews Correlation Coefficient (MCC) as shown in Eq. 2 offers a balanced measure that considers all confusion matrix categories and is robust to class imbalances, making it particularly valuable for small datasets. MCC ranges from -1 (perfect misclassification) to 1 (perfect classification) and provides a more informative evaluation compared to $F_1$-score and balanced accuracy [45].

2 $$\begin{aligned} \text {MCC} = \frac{\text {TP}\cdot \text {TN}-\text {FP}\cdot \text {FN}}{\sqrt{(\text {TP} + \text {FP}) \cdot (\text {TP} + \text {FN}) \cdot (\text {TN} + \text {FP}) \cdot (\text {TN} + \text {FN})}} \end{aligned}$$

## Results

We first report the results of the data preprocessing steps, focusing on the batch effect, and respectively present the experimental results for the classification prediction tasks for $A\beta ^-T^-$ to $A\beta ^+T^+$ using ventricular data. Next, we describe the predictive biomarkers identified by the machine learning models, including both novel and established biomarkers, and discuss them in the context of the current literature.

### Batch Effect on Predictive Results

**Fig. 3:**
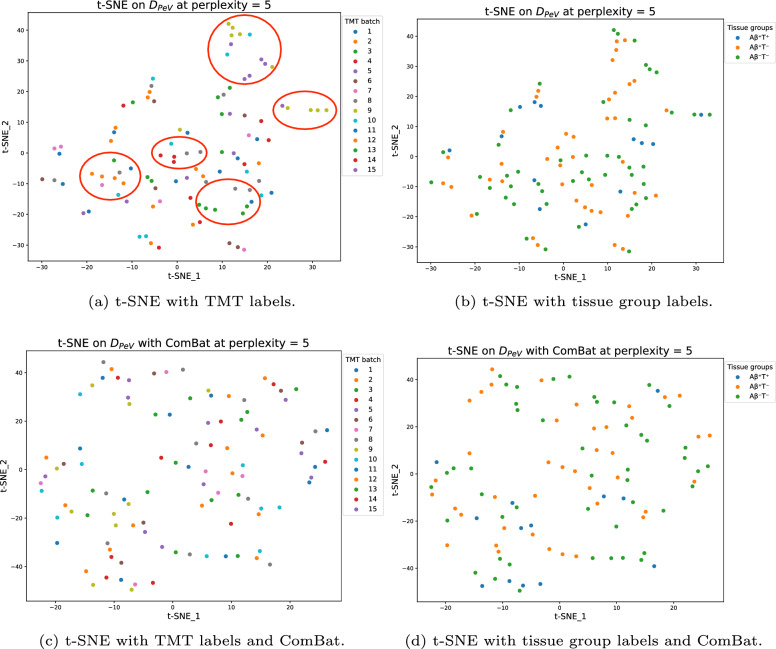
Four t-SNE plots of the $D_{PeV}$ dataset with all features with missing values removed. Figure 3a and c are colored by the TMT batch, while Fig. 3b and d are colored by tissue group. In Figs. 3a and b, ${D}_{PeV}$ has not undergone ComBat batch effect removal. Noticeable clusters in Fig. 3a, as shown with red circles, indicate the presence of batch effect bias. After applying ComBat to ${D}_{PeV}$, Fig. 3c shows increased entropy while retaining similar clustering patterns in the tissue group plot

Dimensionality reduction visualizations using t-SNE reveal distinct TMT batch clusters within the data. We compare the clusters on TMT batch and tissue groups before and after applying ComBat (for details, see Sect. Methods) and see differences. However, the presence of batch effect does not necessarily correlate to poorer clustering of tissue groups (see Fig. 3).

We examine the impact of batch effects on machine-learning models by predicting the TMT batch for each sample using a soft-voting ensemble of LR, RF, and XGB. Without applying ComBat to the dataset, the models achieved accuracies of 55% and 77% in identifying the correct batch among the 15 batches. However, after applying ComBat, model performance significantly declined to 1% and 11% accuracy, indicating the effectiveness of ComBat in mitigating batch effects (for an overview, see Appendix 8). This pattern holds for both minimum and multiple imputations, reinforcing the presence of the batch effect and ComBat’s ability to address it. We observe that ComBat does not appear to influence the prediction of tissue groups, suggesting that batch effect removal might not be necessary.

### Empirical results for tissue groups

In Table 3, we respectively report the performance of the LR, RF, and XGB that predict the diagnosis change from $A\beta ^-T^-$ to $A\beta ^+T^+$ using protein ventricular data (task A2). The results show the average over 10 iterations, 5-fold cross-validated accuracy, $F_1$-score, AUC, and MCC with their 95% confidence intervals.

The best-performing model where all features with missingness were removed, data augmentation through SMOTE so that both classes had an equal amount of samples and feature selection through RFE() until *k* = 2 achieved an AUC of 0.84 (± 0.03).

**Table 3:** Predictive performance comparison of machine learning models on protein ventricular dataset

Model	Accuracy	$F_1$-score	AUC	MCC
Task A2				
XGB	0.80 (± 0.03)	0.55 (± 0.05)	0.81 (± 0.03)	0.43 (± 0.07)
LR	0.76 (± 0.02)	0.53 (± 0.04)	0.80 (± 0.03)	0.39 (± 0.05)
RF	0.80 (± 0.01)	0.58 (± 0.03)	0.84 (± 0.03)	0.46 (± 0.04)
Soft ensemble	0.81 (± 0.02)	0.58 (± 0.03)	0.84 (± 0.02)	0.46 (± 0.045)
Hard ensemble	0.81 (± 0.02)	0.58 (± 0.05)	0.84 (± 0.02)	0.445 (± 0.06)

This table presents the performance of five machine learning models on Task A2, using protein ventricular data. Metrics include accuracy, $F_1$-score, AUC, and MCC, each with 95% confidence intervals

Additional predictive performance results are shown in Appendix 9 for peptide and protein data from the lumbar data. Across all tasks (task A1/2 and task B1/2), we see that despite the statistical advantages in recovering the true values, multiple imputation is not leading to better predictive performance for the binary classification from $A\beta ^-T^-$ to $A\beta ^+T^+$ tissue group.

### Biomarker analysis

To compare the biopsy tissue groups, Kruskal-Wallis tests were performed on the predictive proteins and peptides found in both lumbar and ventricular CSF datasets along with box plots. If statistical significance ($\text {p} < 0.05$) in protein or peptide abundance was achieved between biopsy tissue groups, post-hoc Dunn tests were performed to determine specific differences between pairs of tissue groups. Four established biomarkers that are elevated or decreased during neurodegenerative diseases were also considered during staging. We aim to investigate the statistical significance of the biomarkers we suggest in this work.

#### Established biomarkers

To verify data quality and preprocessing, we examined group differences for four CSF proteins that are well-known to change in abundance in AD: neurofilament light polypeptide (NEFL) [48–50], 14-3-3 protein gamma (YWHAG) [51, 52], neuronal pentraxin-2 (NPTX2) [53, 54] and fatty acid-binding protein - heart (FABP3) [55, 56].

**Fig. 4:**
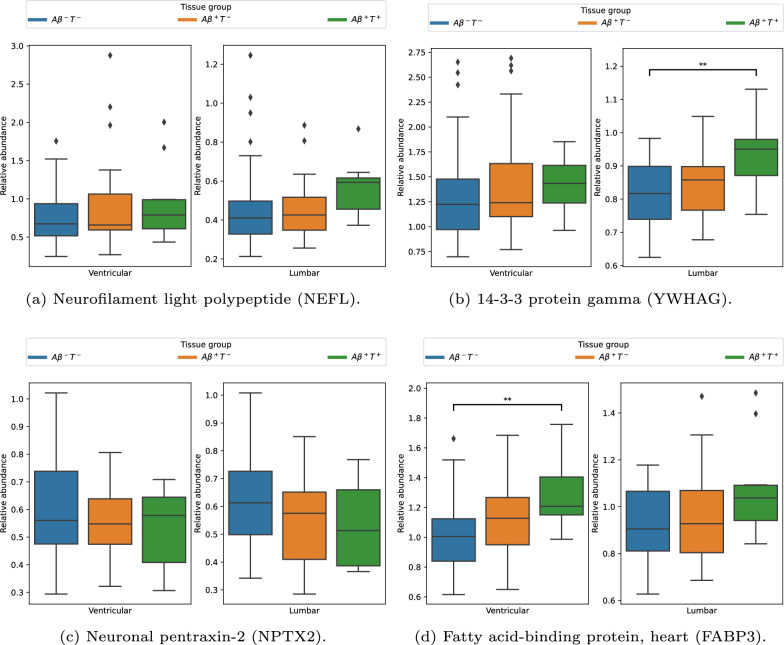
Abundance distribution of proteins on tissue groupings and CSF sample type. The bars on the left in each figure are ventricular CSF, and those on the right are lumbar CSF. Blue bars represent abundance in the $A\beta ^-T^-$ tissue group, orange in $A\beta ^+T^-$, and green in $A\beta ^+T^+$

The distribution of protein abundance across tissue groups and CSF sample types is illustrated in Fig. 4. Table 4 summarizes the statistical comparisons of biomarkers among tissue groups within the ventricular protein dataset and Table 5 for the lumbar protein dataset. The tables include the mean protein abundance and standard deviation for each group, with statistically significant differences ($\text {p} < 0.05$) according to the Kruskal-Wallis test highlighted in bold. A notable finding in the ventricular subgroup shown in Table 4 is the significant difference in FABP3 protein abundance in group $A\beta ^+T^+$ compared to group $A\beta ^-T^-$. In the lumbar subgroup, the $A\beta ^+T^+$ tissue group was determined to be statistically significantly different from $A\beta ^-T^-$ for the YWHAG protein.

**Table 4:** Biomarker comparison between tissue groups on $D_{PV}$

Protein	Kruskal Wallis	p	Post-hoc
NEFL	0.463	0.793	–
YWHAG	2.451	0.294	–
NPTX2	1.39	0.499	–
FABP3	12.642	**0.002**	$A\beta ^-T^-$ and $A\beta ^+T^+$, p=0.002

	$A\beta ^-T^-$	$A\beta ^+T^-$	$A\beta ^+T^+$
NEFL	$0.76 \pm 0.35$	$0.87 \pm 0.53$	$0.94 \pm 0.49$
YWHAG	$1.31 \pm 0.46$	$1.41 \pm 0.50$	$1.42 \pm 0.27$
NPTX2	$0.60 \pm 0.19$	$0.55 \pm 0.13$	$0.53 \pm 0.14$
FABP3	$1.00 \pm 0.22$	$1.11 \pm 0.25$	$1.28 \pm 0.21$

This table presents the protein abundance of each of the tissue groups expressed as mean ± standard deviation. Statistical significance ($p < 0.05$) is highlighted with bold numbers. FABP3 protein abundance in group $A\beta ^+T^+$ was found to be significantly different from those in group $A\beta ^-T^-$

**Table 5:** Biomarker comparison between tissue groups on $D_{PL}$

Protein	Kruskal Wallis	p	Post-hoc
NEFL	4.739	0.094	–
YWHAG	8.995	**0.011**	$A\beta ^-T^-$ and $A\beta ^+T^+$, p=0.008
NPTX2	3.603	0.165	–
FABP3	5.447	0.066	–

	$A\beta ^-T^-$	$A\beta ^+T^-$	$A\beta ^+T^+$
NEFL	$0.47 \pm 0.23$	$0.45 \pm 0.15$	$0.56 \pm 0.14$
YWHAG	$0.82 \pm 0.10$	$0.84 \pm 0.09$	$0.94 \pm 0.10$
NPTX2	$0.63 \pm 0.17$	$0.55 \pm 0.13$	$0.53 \pm 0.15$
FABP3	$0.91 \pm 0.15$	$0.95 \pm 0.18$	$1.08 \pm 0.20$

This table presents the protein abundance of each of the tissue groups expressed as mean ± standard deviation. Statistical significance ($\text {p} < 0.05$) is highlighted with bold numbers. YWHAG protein abundance in group $A\beta ^+T^+$ was found to be significantly different from those in group $A\beta ^-T^-$

#### Novel biomarkers

This section shows the results from the $D_{PV}$ dataset. The following three proteins were found to be selected in all k-folds during feature selection: Myostatin (MSTN), Glutamic-Oxaloacetic Transaminase 1 (GOT1), Calcium/Calmodulin Dependent Protein Kinase II Gamma (CAMK2G). Only GOT1 showed clear significance, potentially indicating that MSTN and CAMK2G require a combination of protein values for diagnostic certainty. Additional staging results are shown in Appendix A.

**Fig. 5:**
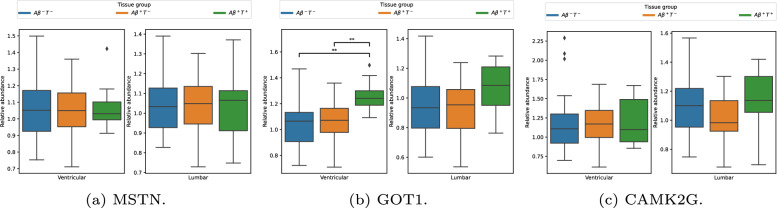
These three protein biomarker candidates are consistently extracted through feature selection in each k-fold. The subfigure captions depict the gene symbol. The proteins descriptions are: 5a - growth differentiation factor 8. 5b - aspartate aminotransferase, cytoplasmic. 5c - calcium/calmodulin-dependent protein kinase type II subunit gamma

**Table 6:** Novel biomarkers across tissue groups on $D_{PV}$

Protein	Kruskal Wallis	p	Post-hoc
MSTN	0.031	0.984	–
GOT1	20.247	**0.00004**	$A\beta ^-T^-$ and $A\beta ^+T^+$, p=0.00002 $A\beta ^+T^-$ and $A\beta ^+T^+$, p=0.0006
CAMK2G	0.89	0.641	–

	$A\beta ^-T^-$	$A\beta ^+T^-$	$A\beta ^+T^+$
MSTN	$1.06 \pm 0.16$	$1.05 \pm 0.16$	$1.07 \pm 0.13$
GOT1	$1.03 \pm 0.16$	$1.07 \pm 0.15$	$1.26 \pm 0.11$
CAMK2G	$1.16 \pm 0.33$	$1.17 \pm 0.25$	$1.20 \pm 0.28$

This table presents the protein abundance of each of the tissue groups expressed as mean ± standard deviation. Statistical significance ($\text {p} < 0.05$) is highlighted with bold numbers. GOT1 protein abundance in group $A\beta ^+T^+$ was found to be significantly different from those in both group $A\beta ^+T^-$ and $A\beta ^-T^-$

The result of the Kruskal-Wallis test highlights that the GOT1 protein shows significant differences across tissue groups, which may be relevant for the classification problem. However, we acknowledge that while the Kruskal-Wallis test identifies statistically significant group differences, it may not directly indicate predictive power for the model. Additionally, the Dunn test revealed that the abundances of the $A\beta ^+T^+$ tissue group were statistically significant compared to both the $A\beta ^+T^-$ and $A\beta ^-T^-$ groups as seen in Table 6. In Fig. 5b it is apparent that the abundance is elevated in the $A\beta ^+T^+$ tissue group.

## Discussion

Next, we discuss the specifics of the data set and the implications on our analysis.

### Impacts of high-dimensional data

High-dimensional data with few samples and high missingness is common in proteomics. There is no universally accepted approach for managing missing values. Striking a balance between removing features with excessive missing data and imputing these values is crucial. Removing features can lead to loss of information, but imputation can introduce biases by distorting feature distributions [57]. Additionally, the method of imputation and level of missingness affects which features are deemed important. By evaluating the impact of removing features with missingness and imputation, our results indicate that any imputation generally performs worse than using only features without missing data.

Our feature selection results suggest significantly reducing the feature space is more beneficial than retaining more features for model training(see Section A). By individually reducing the feature space with four feature-selecting models, we take the union of the models’ feature sets. This approach allowed each model to contribute its strengths to the feature-selection ensemble. In conclusion, we see that the models that perform the best have fewer features and that using all features results in worse performance.

The presence of batch effect in the dataset is clear (see Fig. 3). This conclusion is further strengthened through the prediction of TMT set (see Table 8). However, in most cases, the application of ComBat has minor, negligible, or even negative effects on the result. This leads to the conclusion that the presence of batches in the data has less impact than initially hypothesized.

### Handling of small cohort and feature selection

In addition to the challenges of high-dimensional data, the small cohorts of 51 lumbar and 60 ventricular samples introduce their difficulties, especially with only 10 and 13 samples from the minority class. Splitting these small datasets into training and testing sets can introduce biases or unstable predictions, depending on the split. Instead, we utilize five-fold cross-validation to increase the useable data for training and validation. This further affects the confidence interval, providing more narrow results than without the folds. Additionally, the introduction of k-fold cross-validation complicates the feature selection process. When data is initially split into training and testing sets, feature selection is performed only on the training set. It is, therefore, crucial to perform feature selection within each k-fold to reduce the sample space, not before the data is split in each k-fold. If not, the models risk overfitting the data due to data leakage. This is a common pitfall when working with high-dimensional, small sample-size datasets [36].

Furthermore, extracting features separately in each k-fold ensures that a potential biomarker is stable if present in each fold. If a strong feature is selected in one fold but not in others, it may emphasize outlier samples in the dataset. Therefore, the proposed biomarkers have all been selected in all k-folds, ensuring their applicability to the entire dataset.

### Distinguishing neurodegenerative disorder biomarkers

Within the domain of extracting biomarkers for AD pathogenesis prediction, the dataset used is fairly unique. Typically, studies compare healthy individuals to those with clinically diagnosed AD. Not only does our dataset consist of patients all suffering from iNPH, another prevalent neurodegenerative disorder, but also the diagnosis is pathological. We have shown that some established biomarkers for AD prediction are inadequate for this dataset. The only established biomarker for neurodegenerative disorders that rejected the null hypothesis in a Kruskal-Wallis test was 14-3-3 protein gamma, yet it was not of significant predictive power for the ML models. Therefore, we propose that different biomarkers be used to predict pathological tissue groups for AD in a cohort with iNPH.

Furthermore, the proposed biomarkers differ between lumbar and ventricular CSF. This suggests that there are differences between the sample cohorts and that there may be a need to treat these samples somewhat differently. Only one protein, growth differentiation factor 8, MSTN, is considered a good biomarker for both lumbar and ventricular CSF. In contrast, the other seven proposed protein biomarkers differ between the CSF types. This highlights the fact that protein and peptide abundance fluctuates in the CSF as it traverses through the subarachnoid space, further hinting at a need to establish different biomarkers depending on the space the CSF is extracted from. However, only one protein and three peptides reject the null hypothesis in a Kruskal-Wallis test, indicating significant differences between the groups. These are GOT1 (see Subfigure 5b), PPIB, P23284 159-165 (see Table12), AFM, P43652 215-221 and MAN2A2, P49641 277-283 (see Table  11), but not the other proposed biomarkers. GOT1 is of especial interest, as it is not widely known. It is of further interest as it shows a greater difference in ventricular CSF compared with lumbar, which is not the case for the other, known biomarkers. Direct evidence linking GOT1 to specific pathological mechanisms is currently limited. However, previous research has demonstrated significantly elevated GOT activity in the brains of Alzheimer’s patients [58, 59].Elevated levels of FABP3 have been demonstrated to be associated with an increased likelihood of amyloid pathology [60] . Additionally, higher CSF concentrations of FABP3 have been observed in AD patients compared to individuals with MCI and CN individuals[56].

The resulting insights have diagnostic implications. For one, they suggest that lumbar and ventricular CSF data have different important biomarkers and should be treated differently. The results further emphasize that existing biomarkers for singular neurodegenerative disorders are lacking when differentiating between multiple disorders and that new biomarkers are required when distinguishing between diseases such as iNPH with AD and iNPH without AD. ML models can help identify biomarkers for specific subgroups of cohorts suffering from different neurodegenerative disorders in both lumbar and ventricular CSF.

### Limitations and future work

As mentioned before, a small cohort size of the dataset can lead to potential biases and overfitting, particularly with the few minority-class samples present. Expanding the cohort, especially with more minority-class samples, would reduce the need for synthesizing data through SMOTE. However, a larger cohort introduces risks of batch effects and missing values due to the inclusion of additional TMT batches.

For better generalization in future proteomics research, particularly in the pathological diagnosis of AD using lumbar and ventricular CSF from iNPH patients, several strategies can be explored. Firstly, incorporating multimodal data, such as brain scans of the cohort, would enable more comprehensive comparisons along the AD continuum. Secondly, predicting disease progression rather than just classification could provide more detailed insights. In doing so, adding another biopsy status group called mild cognitive impairment ($A\beta ^+T^-$ in clinical terms) can help achieve this. Thirdly, a more detailed and more diverse cohort can help validate and represent disease progression in stating.

## Conclusion

We studied the problem of detecting AD neuropathology in iNPH patients by proteomic analysis of not only lumbar CSF (standard) but also ventricular CSF (which can only be obtained during surgery to treat iNPH). We treated lumbar and ventricular CSF samples as separate datasets due to their distinct proteomic profiles. Our results indicated that removing features with missing values produced stronger models than imputing them, and the batch effect had minimal impact on the models. No single model consistently outperformed the others; however, while ensemble models were slightly less accurate, they had more consistent confidence intervals in scoring metrics. The best-performing model, a random forest, achieved an AUC of 0.84 (± 0.02) in predicting the change from $A\beta ^-T^-$ to $A\beta ^+T^+$. A comparative analysis highlighted the uniqueness of our dataset, and although being a small sample size, showing a lack of correlation with traditional biomarkers and suggested the need for new proteins and peptides when iNPH is present. We propose eight protein and nine peptide biomarkers to differentiate iNPH patients across the pathological AD spectrum, with one biomarker showing potential in both lumbar and ventricular CSF. Future research should expand the cohort size to allow for other model classes, evaluate the proposed biomarkers, incorporate multimodal data, and conduct longitudinal studies to validate and build on these findings.

## Figures and Tables

**Fig. 6:**
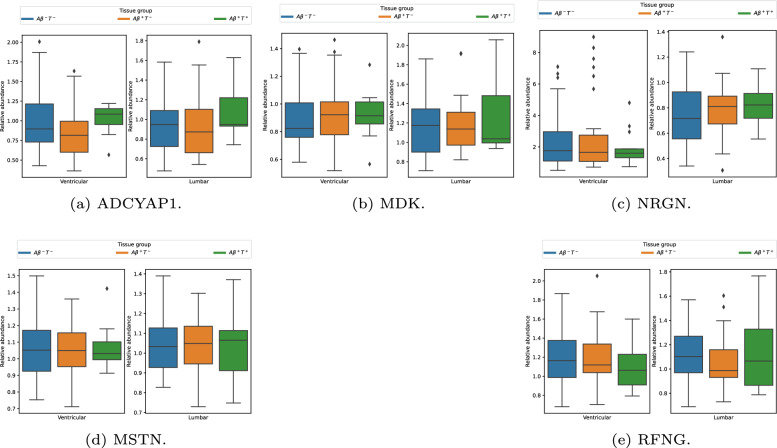
Proposed biomarkers from $D_{PL}$

**Fig. 7:**
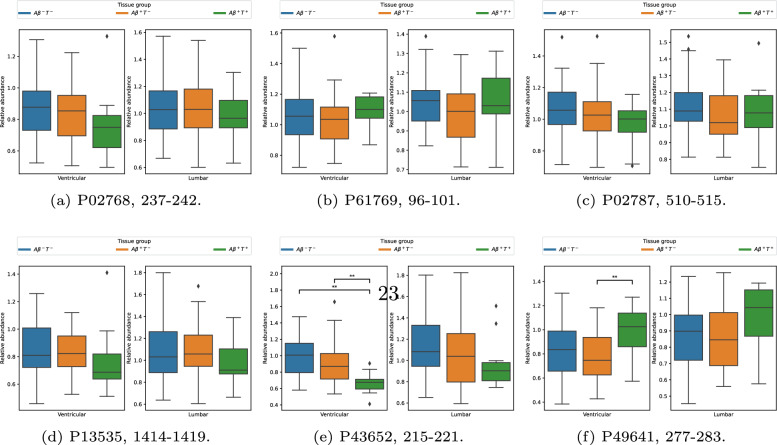
Proposed biomarkers from $D_{PeV}$

**Fig. 8:**
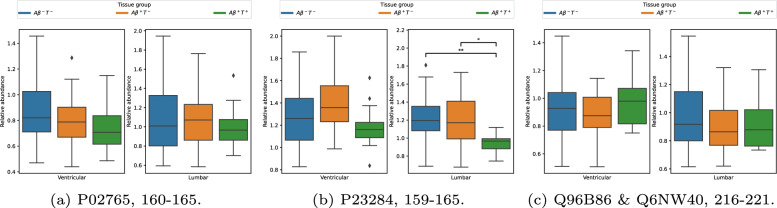
Proposed biomarkers from $D_{PeL}$

## Data Availability

The data used in this article was provided by [24]. The original study aimed to identify prognostic CSF biomarkers for predicting shunt responsiveness in iNPH patients. The datasets used and/or analysed during the current study are available from the authors on reasonable request. Code will become available after acceptance https://github.com/ToffeIO/AD-Biomarkers-Project.

## Appendix A

Biomarker staging

See Figs. 6, 7, 8 and Tables 7, 8, 9, 10, 11 and 12

**Table 7:** Hyperparameters for machine learning models using BayesSearchCV

Model	Hyperparameter	Value
XGBoost	eta max_depth n_jobs n_estimators objective num_classes	Real(0.1, 0.5) Integer(1, 20) -1 Integer(50, 500) multi:softmax 3
Logistic Regression	penalty C solver multi_class max_iter n_jobs l1_ratio	elasticnet Real(0.000001, 100) saga multinomial Integer(1000, 12000) -1 Real(0, 1)
Random Forest	n_estimators max_depth n_jobs min_samples_leaf	Integer(5, 500) Integer(2, 50) -1 Integer(1, 5)

This table lists the hyperparameter ranges for each machine learning model optimized with BayesSearchCV. Tuning options for XGBoost, Logistic Regression, and Random Forest use specific values or ranges (e.g., Real, Integer) to define search spaces, improving model performance by exploring various parameter combinations

**Table 8:** Effect of Imputation, Batch Correction, and Resampling on TMT Set Prediction Accuracy

Dataset	Imputation	ComBat	SMOTE	Soft vote Acc
$D_{PL}$	MI	Off	On	**55%**
$D_{PL}$	MI	On	On	**1%**
$D_{PL}$	Minimum	Off	On	**77%**
$D_{PL}$	Minimum	On	On	**11%**

This table shows soft vote accuracy for predicting outcomes in the TMT set using dataset $D_{PL}$ with different preprocessing strategies. Combinations include imputation methods (Multiple Imputation (MI) and Minimum), batch correction (ComBat on/off), and resampling (SMOTE on/off). Bolded values indicate notable accuracy differences across configurations

**Table 9:** Performance of machine learning models on protein ventricular data for Tasks A1, B1, and B2

	*Accuracy*	$F_1$-score	AUC	MCC
Task A1				
XGB	0.76 (± 0.015)	0.31 (± 0.11)	0.70 (± 0.05)	0.17 (± 0.10)
LR	0.75 (± 0.03)	0.33 (± 0.15)	0.73 (± 0.06)	0.18 (± 0.16)
RF	0.75 (± 0.03)	0.19 (± 0.07)	0.71 (± 0.05)	0.07 (± 0.09)
Soft Ensemble	0.76 (± 0.02)	0.28 (± 0.09)	0.73 (± 0.04)	0.14 (± 0.09)
Hard Ensemble	0.77 (± 0.03)	0.28 (± 0.10)	0.73 (± 0.04)	0.15 (± 0.10)
Task B1				
XGB	0.77 ($\pm 0.04$)	0.35 ($\pm 0.11$)	0.74 ($\pm 0.07$)	0.21 ($\pm 0.13$)
LR	0.77 (± 0.03)	0.37 (± 0.12)	0.66 (± 0.07)	0.23 (± 0.13)
RF	0.79 (± 0.03)	0.39 (± 0.11)	0.74 (± 0.07)	0.27 (± 0.12)
Soft ensemble	0.78 (± 0.04)	0.37 (± 0.13)	0.73 (± 0.07)	0.23 (± 0.14)
Hard ensemble	0.78 (± 0.04)	0.35 (± 0.13)	0.73 (± 0.07)	0.23 (± 0.15)
Task B2				
XGB	0.78 (± 0.03)	0.53 (± 0.07)	0.81 (± 0.05)	0.39 (± 0.09)
LR	0.77 (± 0.03)	0.49 (± 0.07)	0.80 (± 0.05)	0.34 (± 0.07)
RF	0.79 (± 0.03)	0.51 (± 0.07)	0.81 (± 0.04)	0.38 (± 0.09)
Soft ensemble	0.79 (± 0.04)	0.54 (± 0.07)	0.82 (± 0.04)	0.41 (± 0.10)
Hard ensemble	0.79 (± 0.03)	0.54 (± 0.07)	0.82 (± 0.04)	0.41 (± 0.09)

This table shows the predictive performance of the best models on protein ventricular data across tasks A1, B1, and B2. Metrics reported include accuracy, $F_1$-score, AUC, and MCC, each with a 95% confidence interval. Results compare individual models (XGBoost, Logistic Regression, Random Forest) and ensemble methods (Soft and Hard Ensembles), highlighting variations in predictive accuracy and robustness across tasks

**Table 10:** Biomarker comparison between tissue groups on the lumbar protein dataset

Protein	Kruskal Wallis	p	$A\beta ^-T^-$	$A\beta ^+T^-$	$A\beta ^+T^+$	Post-hoc
ADCYAP1	3.02	0.221	$0.93 \pm 0.26$	$0.92 \pm 0.31$	$1.10 \pm 0.29$	–
MDK	0.019	0.99	$1.19 \pm 0.31$	$1.17 \pm 0.27$	$1.23 \pm 0.37$	–
NRGN	1.854	0.396	$0.74 \pm 0.24$	$0.79 \pm 0.20$	$0.81 \pm 0.17$	–
MSTN	0.132	0.93624	$1.05 \pm 0.13$	$1.03 \pm 0.15$	$1.05 \pm 0.19$	–
RFNG	1.915	0.38381	$1.11 \pm 0.22$	$1.05 \pm 0.20$	$1.13 \pm 0.30$	–

This table presents the protein abundance of each of the tissue groups is expressed as mean ± standard deviation. Statistical significance ($\text {p} < 0.05$) is highlighted with bold numbers

**Table 11:** Biomarker comparison between tissue groups on $D_{PeV}$

Peptide	Kruskal Wallis	p	Post-hoc
P02768 [237-242]	5.432	0.066	–
P61769 [96-101]	2.19	0.334	–
P02787 [510-515]	5.204	0.074	–
P13535 [1414-1419]	4.199	0.123	–
P43652 [215-221]	20.429	**0.00004**	$A\beta ^-T^-$ and $A\beta ^+T^+$ p=0.00002 $A\beta ^+T^-$ and $A\beta ^+T^+$ p=0.004
P49641 [277-283]	10.07	**0.007**	$A\beta ^+T^-$ and $A\beta ^+T^+$ p=0.005

	$A\beta ^-T^-$	$A\beta ^+T^-$	$A\beta ^+T^+$
P02768 [237-242]	$0.88 \pm 0.18$	$0.84 \pm 0.17$	$0.76 \pm 0.20$
P61769 [96-101]	$1.05 \pm 0.16$	$1.03 \pm 0.16$	$1.09 \pm 0.10$
P02787 [510-515]	$1.08 \pm 0.15$	$1.03 \pm 0.17$	$0.96 \pm 0.13$
P13535 [1414-1419]	$0.85 \pm 0.20$	$0.82 \pm 0.14$	$0.76 \pm 0.22$
P43652 [215-221]	$0.99 \pm 0.22$	$0.90 \pm 0.25$	$0.66 \pm 0.12$
P49641 [277-283]	$0.82 \pm 0.21$	$0.76 \pm 0.19$	$0.98 \pm 0.20$

This table presents the peptide abundance of each tissue group expressed as mean ± standard deviation. Statistical significance ($\text {p} < 0.05$) is highlighted with bold numbers. The $A\beta ^+T^+$ tissue group showed statistically significant differences in P43652 [215-221] peptide abundance from both $A\beta ^-T^-$ and $A\beta ^+T^-$. Also, the $A\beta ^+T^+$ tissue group was statistically significant from the $A\beta ^+T^+$ tissue group in P49641 [277-283] peptide abundance

**Table 12:** Biomarker comparison between tissue groups on $D_{PeL}$

Peptide	Kruskal Wallis	p	Post-hoc
P02765 [160-165]	0.472	0.79	–
P23284 [159-165]	10.987	**0.004**	$A\beta ^-T^-$ and $A\beta ^+T^+$, p=0.003 $A\beta ^+T^-$ and $A\beta ^+T^+$, p=0.01
Q96B86 & Q6NW40 [216-221]	2.348	0.309	–

	$A\beta ^-T^-$	$A\beta ^+T^-$	$A\beta ^+T^+$
P02765 [160-165]	$1.10 \pm 0.36$	$1.04 \pm 0.27$	$1.00 \pm 0.24$
P23284 [159-165]	$1.20 \pm 0.24$	$1.19 \pm 0.28$	$0.95 \pm 0.10$
Q96B86 & Q6NW40 [216-221]	$0.98 \pm 0.24$	$0.88 \pm 0.16$	$0.92 \pm 0.18$

This table presents the peptide abundance of each tissue group expressed as mean ± standard deviation. Statistical significance ($\text {p} < 0.05$) is highlighted with bold numbers. The P23284 [159-165] peptide was found to have statistically significant differences in abundance in group $A\beta ^+T^+$ compared to $A\beta ^-T^-$ and $A\beta ^+T^-$

## Declarations

### Concent for publication

All authors and co-authors have given their consent for publication.
